# Supplementary material for: Live-Cell Imaging of the Contractile Velocity and Transient Intracellular Ca2+ Fluctuations in Human Stem Cell-Derived Cardiomyocytes
Source: Cells. 2022 Apr 9;11(8):1280. doi: 10.3390/cells11081280 (PMC9031802; doi:10.3390/cells11081280)
Supplement: Supplementary file 1 [file cells-11-01280-s001.zip › Supplementary Material.pdf]

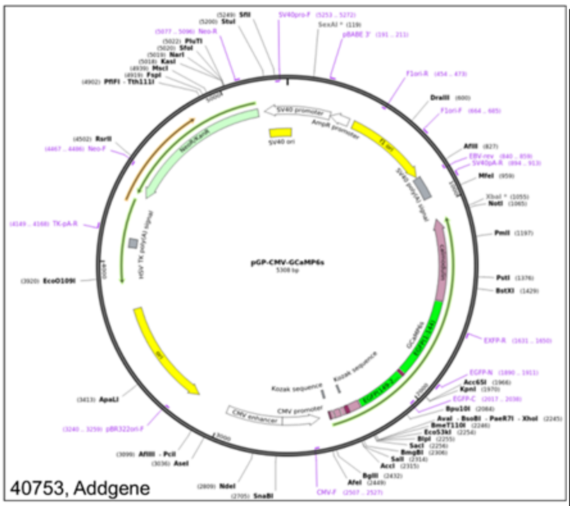

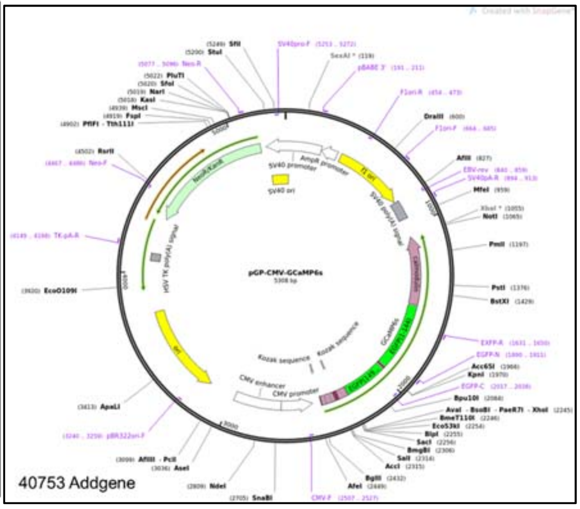

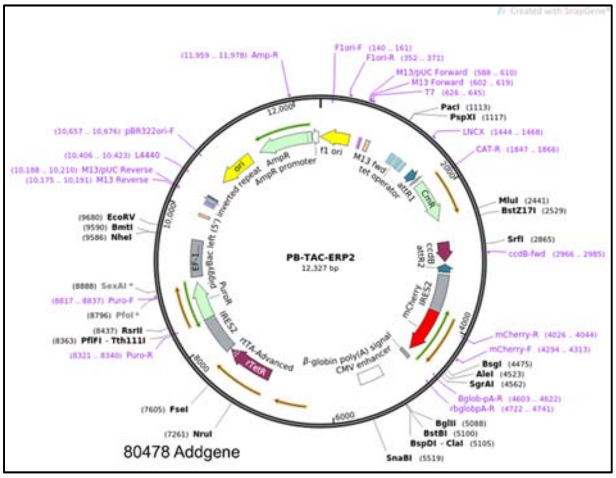

SbfI GCaMP6s

GCaMP6s_attL1_F AGGCTCCTGCAGGACCATGGGTTCTCATCATC

XhoI STOP

GCaMP6s_attL2_R GAAAGCTGGGTCTCGAGCTATCACTTCGCTGTCATC

Universal_attL1_F CCCCGATGAGCAATGCTTTTTTATAATGCCAACTTTGTACAAAAAAGCAGGCTCCTGCAGGCCACCATG

Universal_attL2_R GGGGGATAAGCAATGCTTTCTTATAATGCCAACTTTGTACAAGAAAGCTGGGTCTCGAGCTA

Figure S1. Plasmids required for generation of GECI-eGFP⁺-hiPSCs. GCaMP6 sequence was amplified using the p-GP-CMV-GCaMP6s plasmid (Addgene, Watertown, MA, US) by adding the gateway adapter sequences at both 5' and 3' end as shown below (for detailed description see Figure 1).

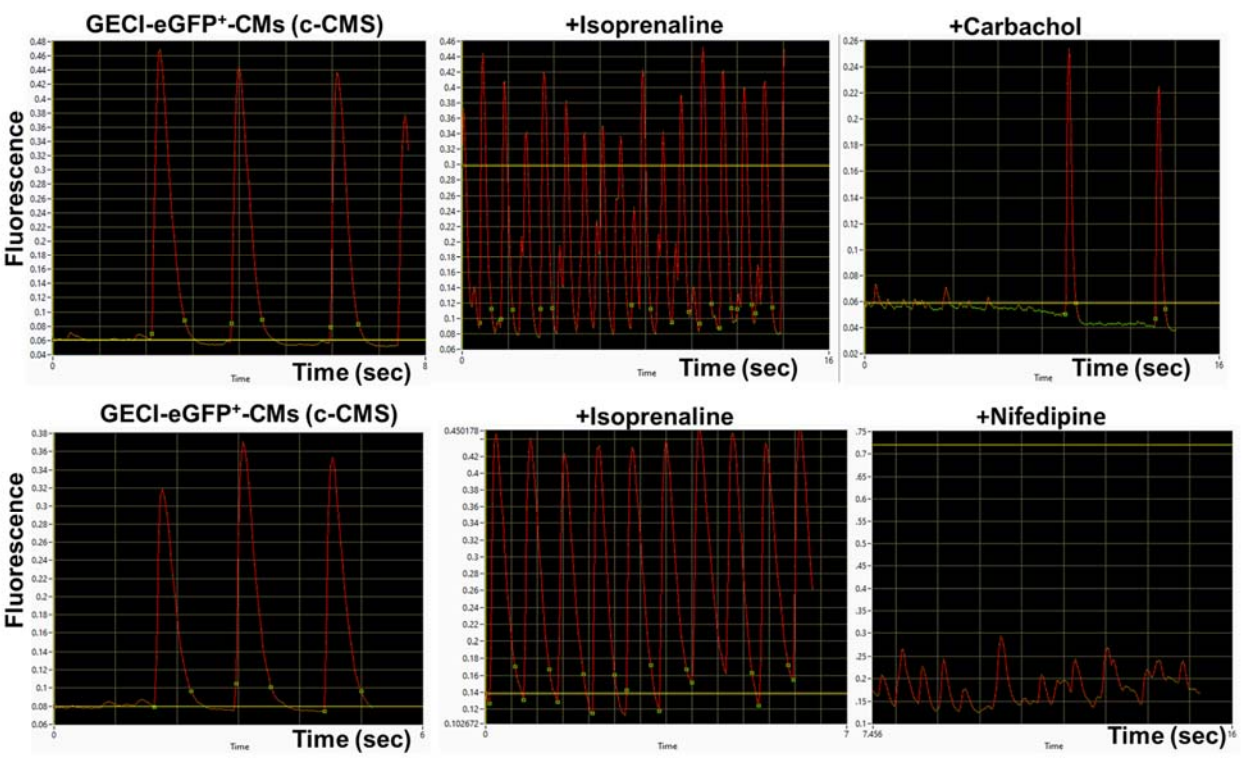

Figure S2. Representative analysis of the different video recordings of the transient changes of intracellular free Ca^{2+} ($[Ca^{2+}]_i$) in GECI-eGFP⁺-CMs by the video analyser 1.9 tool (for more information's see the Material and Methods section).

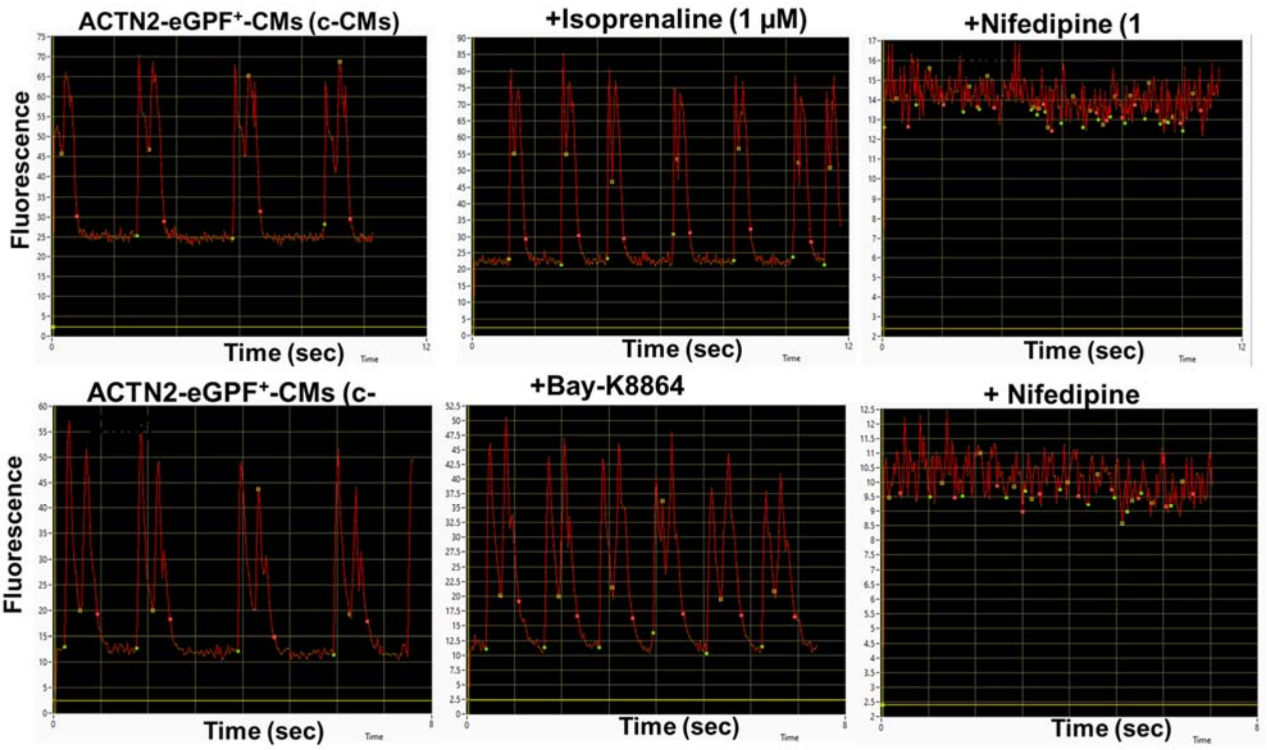

Figure S3. Representative analysis of the different video recordings of the contractile velocity of ACTN2-eGPF⁺-CMs by the video analyser 1.9 tool (for more information's see the Material and Methods section).

Table S1. DNA sequences required for generation of ACTN2-enhanced green fluorescent protein (eGFP⁺)-hiPSC line (IMR90) by the CRISPR-Cas9 and the homology-directed recombination (HDR) approach. DNA sequences of the ACTN2-gRNA with the PAM sequence (underlined). DNA sequences of the donor plasmid with the left and right ~600bp 5' and 3' homology arms (LHA and RHA, respectively) and the Gly-linker.

	**DNA Sequence**
**ACTN2-gRNA**	CGCTTTTATTGCATTCTGAT<u>GGG</u>
**ACTN2-LHA**	GTGATATTTGAGATTTACTTATTTTGACTTTTACCAACCAGACTATTTGGCTGGAATTGTCCTATTTCCCACTGAACT<br>TTTTTTTTAAAAGCTTCATCTTTTCTGGTATGAAATGCAGATCATAGTACGTATCCTCGCATTGTGGCTCAGTTTGAG<br>AACTACTAGTAATGCTCCATTTGCCTTTATGAAGCATATCACCCTTCCTGCTGGCCAGGCCGCTTCTAGATACGCTCC<br>TACAAGTAAAACTTGGCTTTCTGTTGGTTGTCTGGTACTACTATGCCAATAGACTCCCTATTCTTTAGTCCTTTTAAA<br>AAATTAAACAGATGCAAGAAATATGTAAGTATTAAGACTGTTTATGTTGTGGTGTTTCTGCAACTGACTGCAAACACG<br>TGTGTATTTTTTCCCAGCCATACATCCTGGCGGAGGAGCTGCGTCGGGAGCTGCCCCCGGATCAGGCCCAGTACTGCA<br>TCAAGAGGATGCCCGCCTACTCGGGCCCAGGCAGTGTGCCTGGTGCACTGGATTACGCTGCGTTCTCTTCCGCACTCT<br>ACGGGGAGAGCGATCTG
**ACTN2-RHA**	AAGCGGAAGTCACAGTTTGTTTCCTGGAAACTTTGACAAGCTTTATTAAGTTGAGAGAGAGAGAGGGGGAAAAAAAAA<br>GCCTTTCGTAGTTCAGTAATTGCCAGCAATATAACACGGCTAAAATGAAGTTTTTACAGTATATGACATAGTGCGCTT<br>CATAAATAGGTTTATTTCTGAGTTTTTAGCAAAATGTAATGAAATATCAGGTTGATTTCTTTGATTAAACAGAACAAA<br>TTACTTGAGTAATAGGAAATTAGGAGGATCTAGGGACAGAAGGAAAGTGAAAAATGTGAAAATACAAAATACCCAAGA<br>TTTAAGACCGGGGGGAAAAAACCACAAATTGGTAAATAAAGGTTTGCTATTTGTAAAAAATTTCATTTATCTCTAATA<br>TGCTTATGTGATTGGCCCTAGGGGAGTATATTTGGGATTCTAATGTTTTATTTTCATGCTTATCCAAAGATTACTATT<br>GTATCTTCAAATGAATTTAATATTGTGAGATGGAACTG
**Gly-Linker**	GGGGGTGGAGGCTCTGGGGGTGGAGGCTCTGGGGGTGGAGGCTCT
	gRNA sequence with PAM sequence (underlined)
	ACTN2 homology arms, LHA-Left Homology Arm, RHA-Right Homology Arm
	Glycine-rich linker
